# Future novel ecologies: exploring multispecies futures in urban places through a co-design workshop

**DOI:** 10.1007/s11252-025-01846-9

**Published:** 2025-11-13

**Authors:** Mairéad O’Donnell

**Affiliations:** Discipline of Botany, School of Natural Sciences, Trinity College Dublin, University of Dublin, Dublin, Ireland

**Keywords:** Co-design, Multispecies, Workshops, Urban wilding, Transformations, Green spaces

## Abstract

Transforming complex adaptive social-ecological systems, such as urban areas, requires collaboration with stakeholders to address multifaceted challenges. Co-design is a participatory decision-making process during which diverse knowledge and perspectives inform transformative solutions. As urban wilding gains momentum as a strategy for reimagining green space, co-design provides a way to collaboratively explore the values, conflicts, and possibilities that arise when planning for multispecies cohabitation. Urban wilding is an increasingly prominent approach to urban green space design, which emphasises the cohabitation of human and non-human species to foster biodiversity and ecological resilience. To integrate non-human perspectives into urban ecology practice, researchers from the participatory science project NovelEco developed the Future Novel Ecologies co-design workshop, which utilised multispecies methods to incorporate non-human perspectives into the design process. This paper outlines the workshop methodology for planning and conducting a workshop effectively, reflects on the workshop’s effectiveness in fostering collaborative visioning, and suggests adaptations for implementation in alternative settings. Further, the integration of the workshop’s methods into learning environments is discussed to reflect on how they can equip educators, students, and other stakeholders with tools that help reimagine urban ecologies. Insights are also provided about the value of municipalities, planning agencies, and local community groups adopting this workshop to enhance participatory planning and collaborative urban governance. This ecology-with-cities approach advances new inclusive, adaptive urban transformation frameworks by bridging participatory design and ecological, multispecies thinking.

**Supplementary Information:**

The online version contains supplementary material available at 10.1007/s11252-025-01846-9.

## Plain Language Summary

Urban areas are complex places where people, nature, and institutions interact in many ways. Rethinking how we design green spaces in urban areas requires input from different voices and perspectives. One emerging approach, known as urban wilding, focuses on creating spaces where humans and other species can coexist, thereby supporting biodiversity and enhancing resilience.

To explore this approach, researchers from the NovelEco project developed a co-design workshop that brings people together to share knowledge, discuss values, and envision new possibilities. This workshop also explores ways to incorporate the needs of non-human species into the planning process.

This paper explains how the workshop is organised and implemented, and how it may be adapted for use in schools, communities, or by city planners. The reflections show that this workshop can help people think differently about urban green spaces, making planning more inclusive and collaborative. By combining ecological ideas with participatory design, this approach provides educators, municipalities, and communities with practical tools to reimagine urban spaces in more sustainable and inclusive ways.

**Supplementary Information:**

The online version contains supplementary material available at 10.1007/s11252-025-01846-9.

## Introduction

Traditional urban design typically focuses on human needs and economic efficiency, often resulting in spaces that marginalise or exclude non-human life, oversimplify ecological processes, and strengthen extractive relationships with the environment (Connop 2018; Heitlinger and Comber 2018; Zaretsky and Zaretsky 2024). This narrow focus ignores the ecological interdependencies that support all urban life by ignoring the importance of non-human species, disrupting ecosystem functions, and, thus, undermining long-term resilience, ultimately leading to fragmented and unsustainable urban environments (Azadi et al. 2011; Van Patter et al. 2022). Challenging this mindset presents opportunities for transformative change in response to the biodiversity and climate crises (Bulkeley et al. 2021; Hölscher and Frantzeskaki 2021; Nevens et al. 2013; Revi and Rosenzweig 2013).

Framed as complex adaptive social-ecological systems (Bettencourt and West 2010; Moglia et al. 2018; Webb et al. 2018), urban places are dependent on novel ecologies, such as those found in urban wild areas (Gandy 2016; Kowarik 2018; Threlfall and Kendal 2018), which are increasingly vital in urban resilience (Ahern 2016; Collier 2015). Novel ecologies refer to characteristics in ecosystems that have been extensively modified by human influence, often featuring new combinations of species and abiotic conditions that lack any historical precedent (Hobbs et al. 2006). Such unmanaged spaces can be understood through diverse perspectives, including the needs of non-human species (e.g., habitat, resource, and survival requirements) and their ecological roles, to foster cohabitation with humans (Heitlinger et al. 2024; Pineda-Pinto et al. 2023). This aligns with reconciliation ecology (Rosenzweig 2003), which emphasises ‘finding a common ground’ when designing environments for maintaining biodiversity alongside human use. Similarly, urban wilding, an increasingly prominent approach to urban green space design, emphasises the cohabitation of human and non-human species to foster biodiversity and ecological resilience (Ehrnström-Fuentes et al. 2025; Owens and Wolch 2019; Steele 2020).

Taken together, these approaches point toward a broader orientation of multispecies thinking, which explicitly recognises the interconnectedness of humans and non-humans and challenges anthropocentric environmental management (Kennedy 2022). Multispecies thinking views other species as active participants in shaping shared environments, a perspective that becomes particularly salient in urban contexts where social and ecological dynamics interact (Clarke et al. 2019). Incorporating multispecies thinking into the design of urban green spaces extends the notion of collaboration beyond humans, broadens the ethical and ecological scope of planning, and encourages people to consider how other organisms shape and are shaped by planning processes, thereby supporting more inclusive, ethical, and ecologically responsive planning (Van Dooren et al. 2016).

The approach of co-design can bring the ethos of multispecies thinking into formal practice through holistic and participatory methods that incorporate diverse needs and viewpoints into planning and decision-making (Kerr et al. 2023). Co-design provides a promising approach to tackling the complexities of navigating conflicting human and non-human needs; adapting to unpredictable ecological changes; and collaborating across disciplinary, institutional, and community boundaries by promoting inclusive, adaptive, and context-specific planning processes (O’Donnell et al. 2025a; Kerr et al. 2023; Penuel 2019; Romani et al. 2022). Involving diverse stakeholders and integrating non-human roles and needs in design and planning processes may lead to more sustainable urban spaces that meet both ecological and social demands (Fieuw et al. 2022). Recognising these interdependencies creates opportunities for more synthetic and integrated approaches that reflect the full complexity of adaptive social-ecological systems. Thus, co-design can support the conservation and restoration of urban wild spaces, particularly given the growing urgency caused by climate-related challenges, such as extreme rainfall and heatwaves (Lin et al. 2021).

**Fig. 1:**
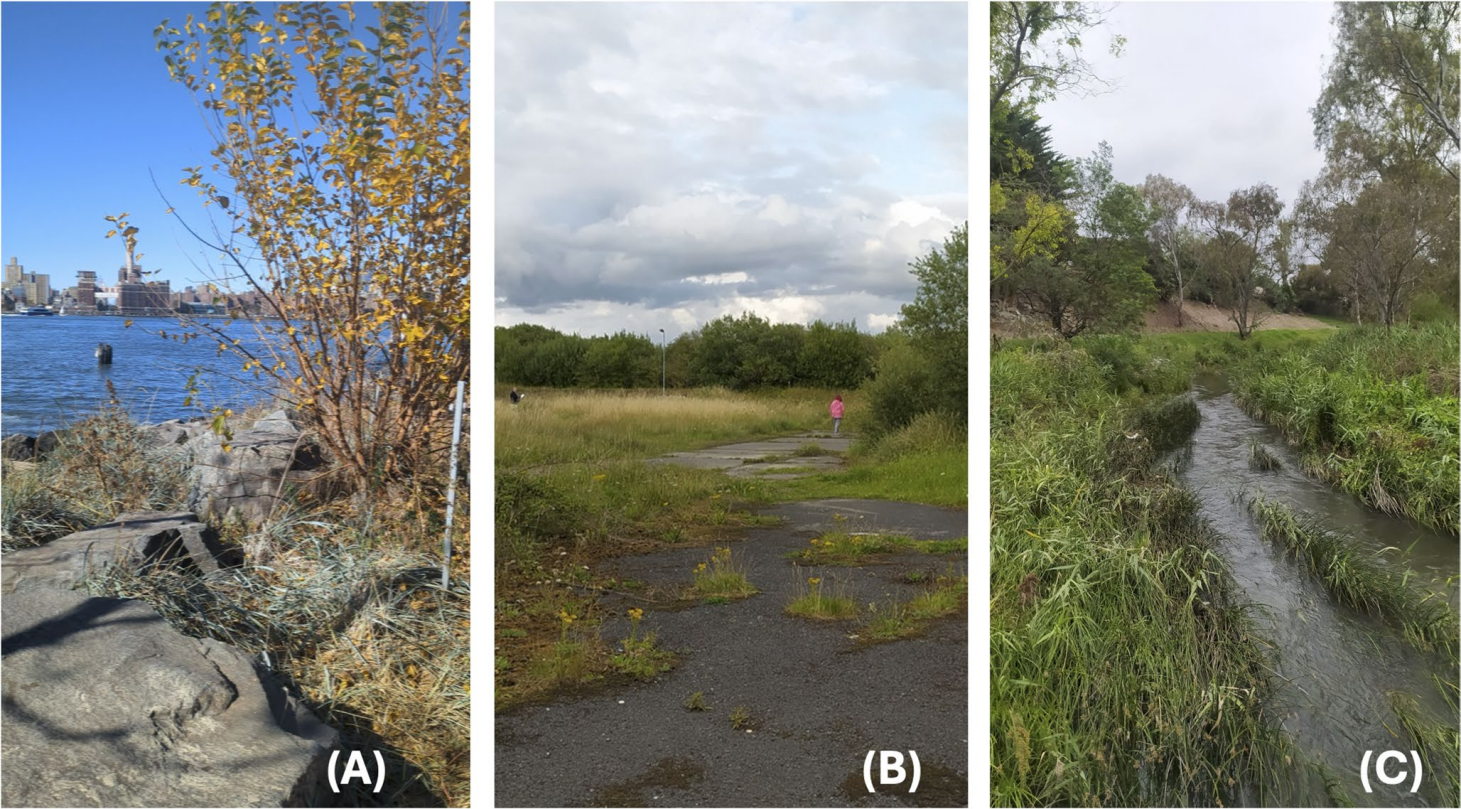
Examples of urban wild spaces where Future Novel Ecologies workshops were conducted: (**A**) an unmanaged coastal verge in New York City, USA; (**B**) an urban meadow in Sallins, Ireland; and (**C**) a verge along a river corridor in Melbourne, Australia. Photos by M. O’Donnell

In this sense, co-design may be viewed as an example of an ecology-with-cities approach that integrates research, teaching, outreach, and community engagement to deepen understanding of urban social-ecological systems and promote more just, resilient, and sustainable cities (Byrne 2022). To explore how a co-design and ecology-with-cities approach could be enacted together in practice, the Future Novel Ecologies (FNE) workshop was developed by Dr Meliss Pineda-Pinto and Dr Mairéad O’Donnell as part of the European Research Council-funded NovelEco research project based at Trinity College Dublin (Ireland). The goal of creating the FNE workshop was to centre human and non-human perspectives, challenging conventional planning paradigms and expanding decision-making to support biodiversity, resilience, and social connection. As part of the project development, eleven workshops were conducted between April 2023 and November 2024 (Table 1) in various urban wild spaces in Australia, Ireland, and the USA (Fig. 1) (Pineda-Pinto et al. 2025). Through embodied sensory and emotional engagement with urban nature (Pink 2015), participants envisioned multispecies futures that reimagine urban spaces (Nijs et al. 2020) as habitats of social-ecological interdependence, flourishing, and justice. To help others plan and implement an FNE workshop, this paper outlines its structure, reflects on its effectiveness for fostering shared envisioning among diverse stakeholders, and discusses lessons learned from the author’s experiences in leading the workshops. The paper also proposes strategies for adapting this approach across classrooms, municipalities, and community groups to help equip people with tools for participatory planning and collaborative governance that support reimagining urban systems. This co-design FNE workshop provides a scalable approach to promoting urban transformations towards more inclusive, adaptable, and ecologically resilient multispecies futures by incorporating the needs and roles of both human and non-human participants.

**Table 1:** The workshop locations, participant groups, site typology and gatekeeper organisation that helped organise the eleven FNE workshops. Participants were grouped into the following communities: COP (Community of Practice), EC (Epistemic Community), and IG (Interest Group)

Location	Participants	Site Typology	Gatekeeper Organisation
Tipperary, Ireland	3 x COP, 1 x EC, 3 x IG	Vacant lot adjacent to carpark	Cahir Tidy Towns*
New York City, USA	3 x COP, 3 x EC, 2 x IG	Vacant lot next to skyrise buildings	Solar 1**
Dublin, Ireland	15 x EC	Wild site collectively cared for by NCAD staff and students	National College of Art and Design (NCAD)
Kildare, Ireland	3 x COP, 5 x IG	Urban meadow along river corridor	Sallins Biodiversity Group
Dublin, Ireland	4 x COP, 5 x EC, 3 x IG	Verge on university campus	University College Dublin
Melbourne, Australia	3 x COP, 7 x EC	Verge along railway line	Swinburne University of Technology
Melbourne, Australia	2 x COP, 3 x EC, 2 x IG	Verge along river corridor	Yarra Riverkeeper Association
Melbourne, Australia	3 x COP, 4 x IG	Verge along river corridor	Friends of Kororoit Creek
New York City, USA	8 x COP, 4 x EC, 1 x IG	Unmanaged park margin	North Brooklyn Parks Alliance
New York City, USA	2 x COP, 2 x EC, 3 x IG	Unmanaged park margin	North Brooklyn Parks Alliance
New York City, USA	2 x COP, 3 x EC, 2 x IG	Unmanaged coastal verge	North Brooklyn Parks Alliance

* A volunteer community group dedicated to improving their local environment for the national Tidy Towns competition in Ireland.** A nonprofit organisation committed to developing and delivering innovative education, training, and technical assistance in New York City

### Overview of the future novel ecologies workshop

Through experiential learning and shared experiences, the FNE workshop aims to foster transdisciplinary dialogue, bringing together participants from diverse backgrounds, including academic researchers, practitioners, and community members, to co-design socially and ecologically inclusive urban spaces. The approach employs co-design methodologies that are grounded in a social-ecological traits framework (Andersson et al. 2021), which provides a common language for engaging with ecological knowledge that resonates with participants’ lived experiences. This framing supports the exploration of interconnected life histories and complex relationships among species by guiding reflections on reciprocity, care, and cohabitation in biodiversity practices, rather than presenting nature through exploitative and utilitarian terms. The workshop structure features an iterative cycle of exploration, discussion, and feedback following each of three activities, allowing participants to revisit earlier ideas, respond to new insights, and collaboratively refine their understanding of multispecies relationships and design possibilities.

The primary foci of the workshop are to address the needs of both human and non-human communities in urban spaces and to foster environments that support ecological resilience and mutual flourishing. Rather than emphasising species richness in numerical terms, the workshop aims to explore conditions where interdependent species support the health and resilience of urban social–ecological systems. However, this approach also involves addressing the uncomfortable realities of multispecies justice, especially concerning species often seen as pests or intruders in urban settings. Species such as rats, pigeons, or possums can play ambiguous roles as ecologically integral organisms who are frequently excluded from visions of fair or desirable multispecies futures. This tension reveals the limitations of inclusion in multispecies design and encourages participants to confront their assumptions and discomforts (Chao 2021; Metzger and Hillier 2024; Power 2009). By highlighting these tensions, the workshop prompts reflection on whether and how urban design can reframe relationships with ‘wild’ species to consider both ‘welcome’ species alongside those who are ‘unwelcome’ because their presence is politically or emotionally contentious, which people are often socially conditioned to repel or eliminate. Pineda-Pinto et al. (2025) provide a more detailed discussion of how previously offered FNE workshops explored these challenges and their significance for multispecies justice.

As part of planning a workshop, participants are identified, selected and invited through local gatekeeper organisations, typically based on their active engagement with urban planning, ecological education, and community-led stewardship. Engaging a diverse range of stakeholders promotes more balanced decision-making by leveraging the strengths of different groups. However, participation in workshops should not be limited to individuals who fit neatly into predefined categories. It is essential to actively include underrepresented and hard-to-reach community members, such as those from Indigenous groups and people with varying backgrounds, interests, and skills, to ensure a fairer and more inclusive process.

**Fig. 2:**
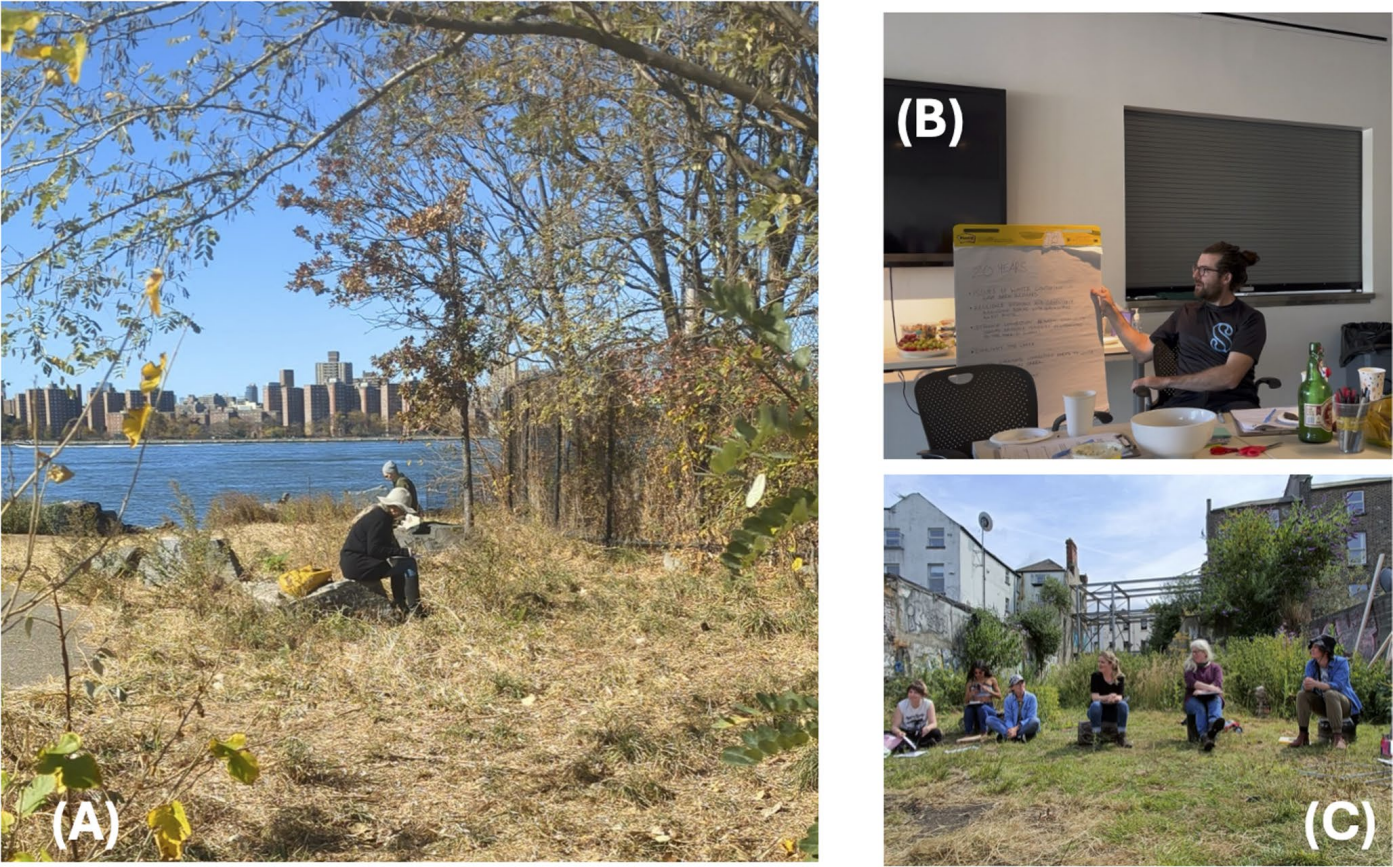
Participants engaged in three activities during the workshops: (**A**) ‘noticing’; (**B**) storytelling; and (**C**) role-playing. Photos by M. O’Donnell (**A**), C. Pierson (**B**), and M. Collier (**C**)

The workshop lasts approximately 2–2.5 h, beginning with a 15-minute introduction to the concept of urban wild spaces (as discussed in Gandy 2016; Kowarik 2018; Threlfall and Kendal 2018) followed by three main activities, each lasting approximately 20 min (Fig. 2; see supplemental file for a detailed description of the workshop structure, activities, resources, and prompts). These activities were selected based on a literature review of co-design methods and the goal of integrating multispecies perspectives (O’Donnell et al. 2025a). After the introduction, facilitators lead participants to an urban wild space, where they undertake a solo walk for approximately 20 min. During this time, participants are encouraged to practice ‘noticing’ (Biggs et al. 2021; Poikolainen Rosén et al. 2022; Tsing 2015) and to use participatory science tools to identify species and reflect on ecological interactions. This is followed by a ~ 10-minute group discussion examining opportunities and challenges for multispecies cohabitation. In the second activity, participants collaboratively develop speculative stories by imagining the site as a thriving multispecies ecosystem (Gonsalves et al. 2023; Nijs et al. 2020; Romani et al. 2022). These narratives, based on their field observations, aim to explore future scenarios of cohabitation, management, and wild autonomy. The third activity involves a role-playing exercise during which participants embody both human and non-human species identified during the walk (Taboada et al. 2024). Through guided discussions and prompts, participants examine interdependencies, threats, and potential collaborations across species, culminating in the sharing of proposals for real-world actions that support mutual flourishing. Finally, facilitators offer a synthesis of key themes, tensions, and opportunities that emerged throughout the workshop, serving as a reflection on the workshop’s collective learning and as a prompt for final contributions. During the workshop, it is essential to allocate sufficient time for discussions, approximately 5–10 min between exercises, and a final 10–20 min for group reflection at the end.

### Planning, preparation, and implementation for inclusive and effective participation

Careful and deliberate planning is vital for ensuring the workshop is effective, inclusive, and grounded in the ecological and social context of its place. To achieve these outcomes, workshop planning involves identifying relevant stakeholders, documenting their relationships to the site, and inviting a diverse group of participants who may contribute varied perspectives to the co-design process. To aid this planning stage, it is recommended that a shared document be established for those planning the workshops to facilitate collaboration and coordination. The following list provides a brief guide for planning and executing the workshops. While the list offers numbered steps, in reality, this is an iterative process, and the steps may change order in practice. Expanded preparatory materials and workshop handouts (including prompts to be used during group discussions) are included in the online supplemental file.

1. Engage a local gatekeeper organisation to build trust and contextual relevance: Collaborate with local organisations that have established relationships and credibility within the area. These gatekeepers may facilitate introductions, help identify suitable participants, offer cultural and ecological insights, and support communication that respects local values and knowledge.
2. Create a shared online folder for collaborating on planning the logistical details: A working document or folder shared with gatekeepers and other facilitators should (1) describe the location and objective of the workshop, (2) list and assign tasks such as booking the venue and catering (if food is being provided), (3) provide a contact list of potential participants and an invitation letter to send to them, and (4) collate workshop resources (e.g., schedule, consent forms, pre-workshop readings; see supplemental file for more suggested resources).
3. Identify a location and conduct a site assessment: Potential locations can include community gardens, vacant lots, school grounds, or small urban wild spaces, all of which are often overlooked but are suitable for meeting the workshop’s objectives. Although there is no strict minimum size, the space should comfortably fit a group of twelve people (or more, depending on the number of participants). Obtain necessary permissions from landowners or relevant authorities to access the site. Visit the location in advance to assess the spatial layout, accessibility, safety, and suitability for the planned activities.
4. Identify and invite diverse stakeholders: It is recommended to include participants representing a range of communities: communities of practice (e.g., urban planners, designers), epistemic communities (e.g., academics, policymakers), interest groups (e.g., residents, community groups), and underrepresented groups. Although these categories often overlap, their combination enriches the design process, mirroring the co-production of place and situated knowledge (Pickett et al. 2022; Home and Bauer 2022). Participants from communities of practice can contribute hands-on expertise. In contrast, those from epistemic communities provide evidence-based insights for policy, and others from interest groups express the values, needs, and preferences of specific societal segments (Wagner et al. 2019). Workshops can also be adapted for children; however, additional ethical approval may be necessary from the relevant institutional ethical committee and, where applicable, participating schools or guardians. To foster multispecies recognition within the activities, participants with biological knowledge about important organism groups should be invited so that they may contribute to discussions about the needs of various species. Finally, sharing electronic calendar invites with attendees helps track attendance and allows them to set reminders in their personal calendars.
5. Plan for group sizes: Aim to form groups of eight to twelve participants that balance diverse perspectives with manageable facilitation, supported by at least two facilitators per workshop. Facilitators may be part of the team organising the workshops, assistants familiar with the organiser’s work, or external co-design practitioners engaged to support the delivery of the workshops. If group sizes exceed twelve, increase the number of facilitators and allocate additional time for discussions to maintain high-quality engagement and ensure that all voices can be heard.
6. Define and communicate the facilitator roles clearly: Facilitators should actively guide discussions, foster inclusivity, attend to participant needs, and document insights thoroughly. Recommendations for reflective facilitation are discussed further in the section ‘Envisioning Urban Ecologies: Lessons Learned’. If workshops are being recorded for research purposes, organisers should prepare recording materials for facilitators, such as notebooks and pens, as well as voice or video recording equipment.
7. Arrange for access to a nearby indoor facility: Secure an accessible indoor space close to the outdoor site to provide a contingency for adverse weather or participant accessibility needs. If possible, the indoor venue should be adjacent or close to the outdoor site to minimise disruptions, support seamless transitions between activities, and accommodate participants’ mobility needs.
8. Organise and distribute the workshop information and materials: Draft workshop information to share with participants via email. Share directions to the site, activity descriptions, and the agenda with participants beforehand to encourage informed and equitable engagement. Additionally, share any relevant readings, government planning documents, or maps related to the location that may inform the workshop discussions. Workshop materials should also be prepared in advance. These include workshop handouts (see supplemental file) with clipboards and pencils for the walk, large drawing sheets (such as poster or flipchart-sized paper) with art supplies (such as markers) for the collaborative storytelling exercise, and name tags for the role-playing activity.

### Envisioning urban ecologies: lessons learned

Based on the author’s experiences, participants of the FNE workshops demonstrated that they could engage with complex ideas from fresh viewpoints, reflect on collaboration methods, explore engagement strategies, and begin to consider policy directions that include multispecies futures. The three workshop activities successfully facilitated dialogue among diverse stakeholders, promoting mutual learning and a holistic understanding of urban ecosystems (e.g., the interdependencies of human and non-human inhabitants). Reflective discussions revealed overlooked biodiversity in vacant lots, while speculative prompts (see the supplemental file for the workshop handout) encouraged participants to consider the needs of non-human species in future urban landscapes. As Aisher and Damodaran (2016) describe, this engagement fosters perceptual shifts, moving participants from anthropocentric to relational multispecies thinking. Rather than seeing nature as a backdrop or resource for human needs, this shift encourages participants to recognise humans as one of many interconnected beings entangled in shared urban environments (Graham 2024). These shifts may lead to practical changes if participants receive support to act on their insights, such as community members initiating stewardship efforts or municipal staff incorporating multispecies considerations into policy and green infrastructure design. However, it is essential to note that municipal staff often face institutional constraints and barriers that limit such collaboration (Kamols et al. 2021; Lodato and DiSalvo 2018). As Berkhout et al. (2003) argue, social learning requires enabling structures, i.e. institutional and organisational arrangements that create the conditions for collaboration, knowledge exchange and sustained action. Thus, the workshop must align with larger planning and governance frameworks that provide ongoing engagement, resources, and decision-making authority.

Facilitation by the coordinators proved to be a vital aspect of the process, as they played a key role in helping participants understand complex ecological concepts, engage in multispecies role-play, and co-develop shared visions. In a workshop setting, facilitation serves as a form of interactional expertise, where coordinators support the exchange of knowledge across disciplines, species, and personal experiences (Cravens et al. 2022). Such effective facilitation requires leaders to possess both ecological literacy and social skills. This includes a working knowledge of local species, ecological relationships, and site-specific environmental histories, alongside the ability to manage group dynamics, navigate differing values, and foster inclusive, respectful dialogue. Literature about how this may be achieved is included in ‘Background Reading’ in the supplemental file. Skilled facilitators can translate complex ecological ideas into accessible prompts, support participants in exploring unfamiliar perspectives, such as those of non-human species, and guide the group toward shared understanding and collaborative outcomes. The supplemental file includes example prompts to achieve this. When facilitators are not part of the community, collaborating with a local organisation can enhance their understanding of local contexts and social dynamics. Such partnerships help facilitators access place-based knowledge, build credibility with participants through the organisations’ established trust networks, and ensure that workshop activities align with ongoing planning initiatives and community-led efforts.

During the workshop discussions, coordinators should work to integrate multiple perspectives into conversations through reflective facilitation (Balcerak 2017). This approach involves continually reflecting on one’s assumptions, power dynamics, and facilitation choices in real-time. In workshops conducted by team members of the NovelEco research project (Pineda-Pinto et al. 2025), this was achieved by actively listening to participants, modifying prompts based on group dynamics, and creating space for quieter voices or non-dominant viewpoints to be expressed. By being sensitive to both verbal and nonverbal cues, facilitators were able to identify tensions, ask appropriate questions, and adjust the workshop’s flow to foster inclusive and thoughtful engagement. Such facilitation is not neutral; it actively involves creating an inclusive space where various perspectives (e.g. scientific, experiential, artistic) are recognised, explicitly invited into the conversation, and respected. This includes employing facilitation techniques such as open-ended questioning, paraphrasing to clarify meanings, and redirecting dominant voices to ensure quieter participants are heard (Heron 1999). For instance, in one of the previous workshops, when a participant shared a cultural story about a local plant species, the facilitator linked it to earlier ecological observations made by others in the group, integrating different knowledge systems to foster a shared understanding of the site’s significance. This encourages knowledge exchange (Cranston et al. 2015), which may lead to shifts in narratives about urban green space management, e.g., away from stories centred on ecological control toward those emphasising cohabitation through care and stewardship (O’Donnell et al. 2025b, *under revision*).

Familiarity with local knowledge, combined with ecological, social, and political expertise, further enhances facilitators’ ability to link theoretical concepts with practical local challenges (Nkedianye et al. 2009), making the learning experience more relevant and impactful. A facilitator with local knowledge may guide discussions effectively by understanding the community’s cultural ties to the land, the importance of specific plants, and the challenges of preserving green spaces in densely populated areas, thus connecting academic environmental understanding with real community needs and experiences. For example, during past FNE workshops, many local citizens shared knowledge about the site’s past land uses, which is vital information for planning future development.

### Alternative applications and potential modifications

Although the FNE workshop was initially developed for an academic research project, it may be applied to other contexts. Municipalities, planning agencies, and community-based organisations, such as environmental justice groups, neighbourhood associations, or urban ecology non-profits, might adapt the workshop to facilitate public engagement in green infrastructure management, ecological planning, and local capacity-building initiatives. The workshop also offers a flexible, hands-on approach to teaching and participatory decision-making in educational, urban governance, and policy-making contexts. Whether utilised in university classrooms or community programs, the workshop fosters an environment conducive to dialogue, visioning, and collaborative action, enabling diverse groups, including local leaders, residents, students, and children, to co-develop inclusive solutions for ecological restoration, biodiversity conservation, and climate resilience. Consequently, the workshop’s activities can contribute to developing valuable skills in systems thinking, inclusive design, and interpersonal communication, all of which are vital for addressing the complexities and challenges of complex social-ecological urban systems.

Alternative approaches may be required to adapt the workshop to different situations. For instance, if visiting an outdoor space is not feasible, facilitators may incorporate guided meditation or sensory envisioning exercises that utilise recordings of natural soundscapes or videos, e.g. through the use of virtual reality. Furthermore, elements from the outdoors may also be brought indoors to sustain sensory engagement, such as soil samples, rocks, plant materials, and small invertebrates (e.g., worms, isopods, beetles). These adaptations may provide participants with a comparable sense of connection and reflection in indoor environments, such as classrooms and nature centres. Similarly, the storytelling, role-playing, and group discussion components may be adapted to emphasise themes and activities that align more closely with the specific goals of a course, community, organisation, or other contexts.

## Conclusions

Because urban areas face growing environmental challenges, integrative and adaptive strategies in urban ecological design are necessary to collaborate with diverse communities and consider the needs of other species. The Future Novel Ecologies workshop provides an embodied and experiential ecology-with-cities methodology that encourages participants to reconceptualise urban ecologies through direct, reflective, sensory, emotional, and interspecies interactions with urban wild spaces. Rather than relying solely on abstract discourse or top-down planning, this methodology fosters a deeper connection to urban greenspace and biodiversity, illustrated through methods such as ‘noticing’, which help individuals engage more intimately with their environment. Such experiences reveal the functions, histories, and future opportunities of urban social-ecological systems, while highlighting challenges such as accessibility, maintenance, conservation, sustainability, and resilience. By incorporating the workshop’s participatory techniques into education and professional practices, facilitators and community leaders can enhance their adaptive capacities to design inclusive, multispecies urban futures.

While the FNE workshop facilitates knowledge sharing, its impact on policy and planning processes depends on broader institutional support. Insights generated by workshop participants must be embedded within formal urban planning frameworks to drive lasting change. The activities of this workshop should be viewed as part of a larger co-production and co-creation effort aimed at transformative urban change, with sustained engagement extending beyond individual sessions (Pickett et al. 2022; Home and Bauer 2022). The workshop emphasises the significance of collaboration in urban planning and design, promoting a deeper understanding of the social, ecological, and institutional aspects necessary for managing urban green spaces (Gaete Cruz 2023; Healey 1998). It also emphasises the context-dependent nature of collaborative urban design processes (Von Schnurbein et al., 2023), which are necessary to influence policy through iterative, ongoing collaboration among policymakers, practitioners, researchers, and communities.

The reflections outlined in this paper provide actionable insights for practitioners and researchers alike, thus contributing to the body of knowledge regarding participatory design processes for the planning of urban green spaces (Kerr et al. 2023; Emmel et al. 2007). Equipping urban practitioners with tools emphasising multispecies thinking, ecological cohabitation, adaptive design, and inclusive engagement is vital for addressing complex social-ecological challenges. Ecology-with-cities approaches that employ participatory methods, such as the FNE workshop, have the potential to foster innovative and equitable solutions (Byrne 2022), contributing to the reimagining of urban places as co-constructed ecosystems where both human and non-human communities can thrive. Integrating co-design with multispecies thinking may cultivate local stewardship, enhance neighbourhood capacity for environmental action, and promote inclusive, ecologically responsive, adaptive urban social-ecological systems. Embedding such frameworks and practices into professional and civic settings may enhance stakeholder engagement and inform long-term policy and planning initiatives, thereby significantly increasing the potential for meaningful transformation in urban design and governance, and ultimately improving the biodiversity and well-being of urban social-ecological systems.

## Supplementary Information

Below is the link to the electronic supplementary material.

Supplementary Material 1 (DOCX 48.1 KB)

## Data Availability

No datasets were generated or analysed during the current study.
