# Supplementary material for: Future novel ecologies: exploring multispecies futures in urban places through a co-design workshop
Source: Urban Ecosyst. 2025 Nov 13;28(6):234. doi: 10.1007/s11252-025-01846-9 (PMC12612005; doi:10.1007/s11252-025-01846-9)
Supplement: Supplementary file 1 — Supplementary Material 1 (DOCX 48.1 KB) [file 11252_2025_1846_MOESM1_ESM.docx]

Supplemental file for “Future Novel Ecologies: Exploring multispecies futures in urban places through a co-design workshop” by Dr Mairéad O’Donnell, published in Urban Ecosystems.

Table of Contents

[A. Background Information and Preparatory Materials 1](#_Toc210825468)

[B. Workshop Handout (with three parts across four pages) 4](#_Toc210825469)

[C. Details of Workshop Methods 8](#_Toc210825470)

[References 11](#_Toc210825471)

# Background Information and Preparatory Materials

The following sections outline preparatory materials facilitators should familiarise themselves with and organise before conducting a Future Novel Ecologies workshop.

**1. Familiarise Yourself with Core Concepts:**

**Core Concepts:**

- **Social-ecological systems** (SES) describe the interrelated and interdependent relationships between humans (social systems) and the environments they inhabit (ecological systems). Instead of viewing society and nature as separate entities, SES frameworks emphasise their co-evolution, mutual feedback, and how human decisions and ecological dynamics interact with each other (Fischer et al., 2015). These systems are often described as complex adaptive systems, meaning they are dynamic, nonlinear, and capable of self-organisation in response to disturbances or changes. In an urban setting (Campbell and Gabriel, 2016, Frank et al., 2017), a social-ecological systems understanding underscores the intricate relationships between governance, communities, and ecological processes that influence resilience, sustainability, and equity (Andersson et al., 2021, Folke et al., 2005, Krueger et al., 2022, McPhearson et al., 2022). Such perspectives allow researchers and practitioners to consider multiple scales of change, while emphasising the need for adaptive and collaborative management approaches (Biggs, et al., 2021a, Ostrom, 2009).
- **Urban wilding** refers to the practice of permitting or fostering natural ecological processes to reestablish themselves within urban environments (Bonthoux and Chollet, 2024, Martin, 2022). Instead of strictly regulating nature through design or maintenance, urban wilding welcomes spontaneous vegetation, wildlife habitats, and dynamic ecologies. This approach challenges conventional aesthetic and functional urban planning standards, advocating for biodiversity, resilience, and the reconnection of humans with nature.
- **Co-design** is a collaborative design methodology in which various stakeholders, including community members, planners, scientists, and designers, collectively engage throughout the design process (Cravens et al., 2022, Echaniz et al., 2022, Nkedianye et al., 2009, O’Donnell et al., 2025). This approach transcends mere consultation to facilitate authentic, shared decision-making, integrating diverse perspectives, needs, and forms of knowledge into the development of spaces, services, or systems. In environmental or urban contexts, co-design is instrumental in fostering more inclusive, equitable, and adaptive outcomes.
- **Multispecies thinking** acknowledges the intricate entanglements between humans and a diverse spectrum of non-human life forms, including plants, animals, fungi, and microbes (Celermajer et al., 2022, Locke and Munster, 2015, Rupprecht et al., 2020). These approaches seek to consider these relationships within the realms of research, design, and governance. Instead of relegating non-human entities to mere background elements or resources, multispecies perspectives regard them as active participants and co-inhabitants of shared environments. In urban settings, multispecies approaches strive to promote ecological justice and the mutual flourishing of species.

**2. Understand the Context of Urban Wild Spaces:**

● Review case studies of urban wilding projects demonstrating collaboration and participatory processes, such as local projects focused on river restoration, climate resilience, citizen science, nature-based solutions, and biodiversity conservation. Examples of such projects include Tempelhofer Feld in Berlin (https://www.tempelhoferfeld.de/en/), Rewild My Street in London (https://www.rewildmystreet.org/), and The Field at the National College of Art and Design in Dublin (https://www.ncad.ie/about/ncad-field/).

● Familiarise yourself with local or regional policies, urban planning, and biodiversity challenges. Examples of beneficial documents include local area plans, climate action plans, environmental impact assessments, public consultation reports from urban development projects, and green space management plans relating to the site where workshops will be conducted. Furthermore, 1) identifying threatened species or habitats in your region through local biodiversity inventories or reports, 2) learning about invasive species management programs in your area, and 3) investigating fragmentation of green spaces or loss of pollinator habitats due to urban expansion or poor land-use planning would all be advantageous.

**3. Prepare the Materials:**

● **Pre-Session Informational Materials for Participants:** If available, it is beneficial to provide maps of the local area, GIS datasets such as land use, vegetation cover, and habitat connectivity, as well as local authority resources like zoning maps, green infrastructure strategies, and biodiversity action plans tailored to the workshop location. This allows participants to be better informed about the ecological, spatial, and policy context of the workshop area, including understanding where key habitats are located, how land is currently used, historical and existing plans for development or conservation, and identifying opportunities for intervention or collaboration.

● **Activity Materials:**

○ An example Workshop Handout is supplied below that can be used as is or adapted for your specific needs. This handout is intended as a guiding tool for participants. Facilitators are encouraged to invite participants to engage with the elements of the space that resonate most with them. Participants should be supported in choosing the mode of expression they feel most comfortable with, whether that be writing, drawing, photography, or voice recording.

○ Clipboards with pens and printed prompts to enhance immersion for the walk and practice of ‘noticing’.

○ Writing materials for storytelling and role-playing exercises (e.g., markers, sticky notes, large paper/poster/flipchart, name tags).

○ Online citizen science tools such as SpotterOn or iNaturalist.

● **Stakeholder Profiles:** During the reflections about the walk, facilitators should take notes of the various species mentioned and create profiles to assign to each participant during the role-playing exercise. Try to achieve an even distribution of profiles among mammals, insects, trees, wildflowers, humans, and other species.

**4. Create an Agenda:**

● Design a structured plan with clear time allocations for each activity stage (e.g., introduction, wild walk, storytelling exercise, multispecies roleplaying, group work, feedback). This can be shared with participants during the planning stage.

# Workshop Handout (with three parts across four pages)

**Welcome to the Future Novel Ecologies Workshop!**

We’re excited to have you join us for this collaborative session, which was made possible in partnership with [insert gatekeeper organisation’s name here]. Today’s workshop will be facilitated by [insert information on facilitators and their connection with today’s workshop].

**Purpose of the Workshop**
[Please insert a brief description of the workshop’s overall aim. For example, if the workshop is intended as a learning experience, outline how participation may support understanding or skill development. Describe how participant input will inform or shape the outcomes if the workshop contributes to a design or planning process. This section should clarify why the workshop is taking place and how participants' engagement will be meaningful.]

Over the next 2 to 2.5 hours, we hope to create a fun, relaxing, and interactive environment where you can engage with these ideas.

**Location/Date:** ____________________________

**Name/Species:** _____________________________

**Part 1 – Wild Transect Walking**

As you walk through and experience the site, please consider and take note of some of the suggestions below. Feel free to write them down, draw, sketch, or think about them.

**The overall environment** (e.g., size, climate, soil, contamination and other traces of human activity, etc.)

**Species assemblages:** (e.g., groupings and abundance of plants, birds, mammals, most abundant species)

**Characteristics of plants and animals:** (e.g., notice characteristics such as leaf size and shape, height, and colours that stimulate your senses (e.g., touch, smell, sound, taste) and identify what different species might need to flourish)

**Connections to the space:** (e.g., consider aspects of the space that you are drawn to most and why)

**Barriers and Opportunities:** (e.g., consider benefits and barriers (e.g., impacts, risks) for species to flourish)

**Part 2 – Storytelling**

As a group, create a short story inspired by the transect walk. Reflect on species interactions, past land uses, future impacts, and how you imagine this place in 5/10/100 years. Present this future scenario story for discussion. Feel free to sketch, write, or discuss your ideas.

Don’t forget that the story needs characters (who), time and place (where), events (what happened/happens), temporality (a sense of movement), and a plot (what it’s about/directionality).

**Part 3 – Multi-Species Role-Playing Game**

Each person will be assigned a role. Working as a team, identify ways to help us achieve the future scenario from the previous activity.

To start, each species will introduce itself to the group (who they are, what they do, what they need in their environment, who they connect with in the room, etc.).

**Game Rules:**

1. Identify three actions required to achieve the future scenario you described earlier.

2. The needs of all species must be considered

3. As many species as possible must benefit from these three actions.

4. Opportunities for species to flourish must be enhanced.

5. Trade-offs between species must be equitably considered.

Consider the following questions when identifying ways to reach your desired future:

- - How do the species relate to and benefit from each other?
  - What resources do we need?
  - What must we (the community and individuals) do to get there?
  - Who inside and outside your community should be included to enable this transformation?

# Details of Workshop Methods

The initial workshop activity takes place in an urban wild space, aiming to deepen participants' connection with the environment while fostering a multispecies awareness. Participants begin with a 20-minute solo exploration of the site, during which they are encouraged to identify species, observe ecological interactions, and reflect on the traits that characterise the area (see B. Workshop Handout above for prompts). This process is supported by participatory science tools such as the NovelEco app (https://noveleco.eu/), which facilitates species identification and engagement with the site’s ecological complexity. Walks are an established practice for observing vegetation, interactions, and spatial dynamics within specific ecological contexts (Dhaundiyal and Pant, 2020). Participants are encouraged to approach the site not just as observers but as active listeners, tuned into subtle cues, relationships, and multispecies presences (Biggs et al., 2021b, Biggs et al., 2021c, Poikolainen Rosén et al., 2022, Tsing, 2015). To guide their observations, participants are asked to record specific elements, including environmental features and changes, species identification and groupings, species traits and their related needs or capabilities, and the ecological functions that those species perform. They are also encouraged to reflect on the site’s historical land use, its current state, and potential future directions, including their hopes for its long-term development. This place-based and relational approach offers insights that might be missed by more traditional research methods, enabling participants to uncover complex ecological dynamics and site-specific multispecies interactions (Narayanasamy, 2009). After the solo exploration, participants reconvene to share their observations and discuss potential opportunities and challenges to multispecies mutual flourishing at the site. Multispecies mutual flourishing is described to participants as the shared thriving of human and non-human beings within an ecosystem, where the needs, relationships, and well-being of multiple species are recognised, supported, and maintained over time.

The second workshop activity involves participants collaboratively imagining the urban wild site as a thriving future ecosystem that could support a variety of both human and non-human species. The future scenarios could feature spaces with little human presence, prompting participants to consider whether humans and non-humans may coexist or whether certain areas should be reserved primarily for non-human communities. The exercise also encourages comparisons with more managed urban environments, inviting reflection on how lessons from these wilder spaces could inform urban cohabitation strategies. In smaller groups (~5-8 individuals, depending on overall group size), participants should spend about 20 minutes crafting narratives about how the site might evolve into a place of mutual flourishing among multiple species (Gonsalves et al., 2023, Nijs et al., 2020, Romani et al., 2022). The stories, rooted in observations from the initial walk, aim to turn ecological insights into imaginative futures. Storytelling serves as a participatory method to communicate history, values, cultural meanings, and knowledge, while also fostering creative speculation about what might happen next (Waller, 2003). Throughout this process, participants organise their stories around key elements: characters (who inhabit the space), settings (where the events occur), events (what takes place), temporality (how time unfolds), and the plot (the main message). Afterwards, each group shares their vision of the site’s transformation with the broader audience (facilitators and other groups, if necessary), sparking discussion and reflection on the many ways urban ecologies could be reimagined through a multispecies perspective.

The third activity, which lasts approximately 30 minutes, involves participants in a role-playing exercise designed to deepen their understanding of multispecies needs and interdependencies (Taboada et al., 2024). Each participant is given a role based on a species (or larger taxonomic group) identified during the earlier site exploration. These roles encompass both human stakeholders, such as local developers or groundskeepers, and non-human entities, such as sparrows, ants, mycorrhizal fungi, and urban foxes. Participants wear name tags displaying their assigned organism and then participate in a facilitated round-table discussion. This immersive activity allows participants to temporarily consider aspects from another being's perspective and explore that species’ experiences, dependencies, and challenges within the ecosystem. Creative prompts and guided discussion questions (see the Workshop Handout above) encourage participants to explain the conditions required for their assigned species to survive and thrive, as well as the relationships it relies on. For example, they might be asked: “Who around the table do you connect with?” or “Is there anyone here who poses a threat to your survival, and why?” Where necessary, participants should be encouraged to support one another in filling gaps in their knowledge of species. Similar to multispecies workshops for speculative urban futures (Clarke et al., 2018), the activity reveals shared needs, tensions, and possibilities for cohabitation. It also helps foster empathy across species boundaries, encouraging participants to see urban environments as multispecies spaces shaped by overlapping and sometimes conflicting interests. As participants negotiate, collaborate, and advocate from their assigned roles, they should be encouraged to begin forming a consensus on actions that could support more inclusive urban wilding pathways. This game-based approach relies on the well-established value of role-play as a method for social learning (Craven et al., 2017). By embodying diverse species, both human and non-human, participants broaden their understanding of ecological responsibility and reimagine future urban ecosystems from a more-than-human perspective.

The workshop concludes with a facilitated group discussion, where participants propose distinct, real-world actions aimed at supporting the mutual flourishing of humans and non-humans within urban environments. These proposals are grounded in the insights and perspectives developed throughout the workshop activities, including the walk, storytelling exercises, and role-play sessions. Participants are encouraged to consider how their ideas could be applied in their contexts, and they receive constructive feedback from their peers to help refine and strengthen their proposed actions. Following this, facilitators offer a synthesis of key themes, tensions, and opportunities that had emerged across the sessions. This summary serves as both a reflection on the workshop’s collective learning and a prompt for final contributions. Participants are then invited to share additional thoughts, raise outstanding questions, or suggest ways in which the insights generated might inform broader practices of multispecies design and urban ecological planning. Facilitators should also outline proposed follow-up actions, including the creation of a shared online space where participants could upload and access photos taken during the workshop, revisit any resources discussed, and continue conversations sparked during the session. A shared online document can be made available to collect contact details for those interested in staying connected, receiving updates about future workshops, or participating in related collaborative opportunities.

# References

Andersson, E., Haase, D., Anderson, P., Cortinovis, C., Goodness, J., Kendal, D., Lausch, A., McPhearson, T., Sikorska, D. & Wellmann, T. 2021. What are the traits of a social-ecological system: towards a framework in support of urban sustainability? npj Urban Sustainability*,* 1**,** 14, DOI: 10.1038/s42949-020-00008-4

Biggs, R., Clements, H., de Vos, A., Folke, C., Manyani, A., Maciejewski, K., Martín-López, B., Preiser, R., Selomane, O. & Schlüter, M. (2021a) What are social-ecological systems and social-ecological systems research? The Routledge handbook of research methods for social-ecological systems. Routledge

Biggs, H. R., Bardzell, J. & Bardzell, S. Watching myself watching birds: Abjection, ecological thinking, and posthuman design. Proceedings of the 2021 CHI Conference on Human Factors in Computing Systems, 2021b. 1-16.

Biggs, R., De Vos, A., Preiser, R., Clements, H., Maciejewski, K. & Schlüter, M. (2021c) The Routledge handbook of research methods for social-ecological systems, Taylor & Francis.

Bonthoux, S. and Chollet, S. (2024) Wilding cities for biodiversity and people: a transdisciplinary framework. Biol Rev, 99: 1458-1480. DOI: https://doi.org/10.1111/brv.13076

Campbell, L. K. & Gabriel, N. 2016. Power in urban social-ecological systems: Processes and practices of governance and marginalization. Urban Forestry & Urban Greening, 19**,** 253-254

Celermajer, D., Schlosberg, D., Rickards, L., Stewart-Harawira, M., Thaler, M., Tschakert, P., Verlie, B. & Winter, C. (2022) Multispecies justice: theories, challenges, and a research agenda for environmental politics. Trajectories in environmental politics.

Clarke, R., Heitlinger, S., Foth, M., DiSalvo, C., Light, A. & Forlano, L. More-than-human urban futures: speculative participatory design to avoid ecocidal smart cities. Proceedings of the 15th Participatory Design Conference: Short Papers, Situated Actions, Workshops and Tutorial-Volume 2, 2018. 1-4.

Craven, J., Angarita, H., Perez, G. C. & Vasquez, D. (2017) Development and testing of a river basin management simulation game for integrated management of the Magdalena-Cauca river basin. Environmental modelling & software, 90, 78-88, DOI: <https://doi.org/10.1016/j.envsoft.2017.01.002>.

Cravens, A. E., Jones, M. S., Ngai, C., Zarestky, J. & Love, H. B. (2022) Science facilitation: navigating the intersection of intellectual and interpersonal expertise in scientific collaboration. Humanities and Social Sciences Communications, 9, 256. DOI: https://doi.org/10.1057/s41599-022-01217-1

Dhaundiyal, D. & Pant, R. (2020) It takes a village: Community based participatory research as a design research tool. DISCERN: International Journal of Design for Social Change, Sustainable Innovation and Entrepreneurship, 1, 12-26.

Echaniz, A., Celik, S., and Ham, P. (2022) A participatory approach in urban development: Co-designing resilient coastal neighbourhoods, in Lockton, D., Lenzi, S., Hekkert, P., Oak, A., Sádaba, J., Lloyd, P. (eds.), DRS2022: Bilbao, 25 June - 3 July, Bilbao, Spain. DOI: <https://doi.org/10.21606/drs.2022.573>

Fischer, J., Gardner, T. A., Bennett, E. M., Balvanera, P., Biggs, R., Carpenter, S., Daw, T., Folke, C., Hill, R., Hughes, T. P., Luthe, T., Maass, M., Meacham, M., Norström, A. V., Peterson, G., Queiroz, C., Seppelt, R., Spierenburg, M. & Tenhunen, J. 2015. Advancing sustainability through mainstreaming a social–ecological systems perspective. Current Opinion in Environmental Sustainability, 14**,** 144-149, DOI: https://doi.org/10.1016/j.cosust.2015.06.002

Folke, C., Hahn, T., Olsson, P. & Norberg, J. (2005) Adaptive Governance of Social-Ecological Systems. Annual Review of Environment and Resources, 30, 441-473, DOI: <https://doi.org/10.1146/annurev.energy.30.050504.144511>

Frank, B., Delano, D. & Caniglia, B. S. 2017. Urban systems: A socio-ecological system perspective. Sociology International Journal, 1**,** 00001.

Krueger, E. H., Constantino, S. M., Centeno, M. A., Elmqvist, T., Weber, E. U. & Levin, S. A. 2022. Governing sustainable transformations of urban social-ecological-technological systems. npj Urban Sustainability*,* 2**,** 10, DOI: 10.1038/s42949-022-00053-1.

Locke, P. & Munster, U. (2015) Multispecies ethnography, Oxford University Press Oxford.

Gonsalves, K., Aia-Fa’aleava, A., Thanh Ha, L., Junpiban, N., Narain, N., Foth, M. & Caldwell, G. A. (2023) TransHuman Saunter: Multispecies storytelling in precarious times. Leonardo, 56, 125-132.

Martin, L. J. (2022) Wild by Design: The Rise of Ecological Restoration, Harvard University Press.

McPhearson, T., Cook, E. M., Berbés-Blázquez, M., Cheng, C., Grimm, N. B., Andersson, E., Barbosa, O., Chandler, D. G., Chang, H., Chester, M. V., Childers, D. L., Elser, S. R., Frantzeskaki, N., Grabowski, Z., Groffman, P., Hale, R. L., Iwaniec, D. M., Kabisch, N., Kennedy, C., Markolf, S. A., Matsler, A. M., McPhillips, L. E., Miller, T. R., Muñoz-Erickson, T. A., Rosi, E. & Troxler, T. G. (2022) A social-ecological-technological systems framework for urban ecosystem services. One Earth, 5, 505-518, DOI: <https://doi.org/10.1016/j.oneear.2022.04.007>

Narayanasamy, N. (2009) Participatory rural appraisal: Principles, methods and application, SAGE Publications India.

Nijs, G., Laki, G., Houlstan, R., Slizewicz, G. & Laureyssens, T. Fostering more-than-human imaginaries: Introducing DIY speculative fabulation in civic HCI. Proceedings of the 11th Nordic Conference on Human-Computer Interaction: Shaping Experiences, Shaping Society, 2020. 1-12.

O’Donnell, M., Collier, M., Pineda-Pinto, M., Cooper, C., Nulty, F. & Casteñeda, N. R. (2025) Redefining co-design for social-ecological research and practice: A systematic literature review. Environmental Science & Policy, 164, 103998.<https://doi.org/10.1016/j.envsci.2025.103998>.

Ostrom, E. (2009) A general framework for analyzing sustainability of social-ecological systems. Science, 325, 419-422, DOI: 10.1126/science.1172133

Nkedianye, D., Kaelo, D., Reid, R., Neselle, M., Onetu, L., Makui, O., Said, M., Kiruswa, S., Kristjanson, P. & Kamuaro, O. (2009) Linking Knowledge with Action Using Community Facilitators to Span Boundaries: Lessons from East Africa. CID Graduate Student and Research Fellow Working Paper Series.

Poikolainen Rosén, A., Normark, M. & Wiberg, M. (2022) Towards more-than-human-centred design: Learning from gardening. International Journal of Design, 16, 21-36.

Romani, A., Casnati, F. & Ianniello, A. (2022) Codesign with more-than-humans: toward a meta co-design tool for human-non-human collaborations. European Journal of Futures Research, 10, 17.

Rupprecht, Christoph DD, Joost Vervoort, Chris Berthelsen, Astrid Mangnus, Natalie Osborne, Kyle Thompson, Andrea YF Urushima et al. "Multispecies sustainability." Global Sustainability 3, 2020: e34.

Taboada, M., Turner, J., Seevinck, J. & Foth, M. (2024) A worldbuilding approach for pluriversality in codesign. CoDesign, 20, 218-241.

Tsing, A. L. (2015) The mushroom at the end of the world: On the possibility of life in capitalist ruins, Princeton University Press.

Waller, S. E. (2003) Story-telling and Community Visioning: Tools for Stustainability, Sustainability Policy Unit, Department of the Premier and Cabinet, 2003.
